# Improved oxygen saturation and acclimatization with bacteriotherapy at high altitude

**DOI:** 10.1016/j.isci.2025.112053

**Published:** 2025-02-17

**Authors:** James J. Yu, Esteban A. Moya, Hunter Cheng, Kiana Kaya, Tim Ochoa, Santiago Fassardi, Eli Gruenberg, Alex Spenceley, Pamela DeYoung, Elizabeth V. Young, Laura A. Barnes, Alina Lugo, Ana Sanchez-Azofra, Jeremy E. Orr, Erica C. Heinrich, Atul Malhotra, Tatum S. Simonson

**Affiliations:** 1 Division of Pulmonary, Critical Care, Sleep Medicine, and Physiology, Department of Medicine, University of California, San Diego, La Jolla, CA, USA; 2 Division of Biomedical Sciences, School of Medicine, University of California, Riverside, Riverside, CA, USA

**Keywords:** Pharmacology, Environmental health

## Abstract

High altitude imposes physiological stress on the human body due to reduced oxygen availability, and options to improve acclimatization are limited. Seventeen participants underwent a randomized, doubled-blinded, placebo-controlled study to test the effects of a multi-strain probiotic on acclimatization to high altitude (3,800 m). The primary outcome was oxygen saturation (SpO_2_) during both daytime and nighttime. Secondary measurements included acute mountain sickness (AMS) score, sleep measurements, ventilation, resting heart rate, blood pressure, heart rate variability, and fasting glucose levels. The probiotic group exhibited a higher daytime and nighttime SpO_2_ compared to the placebo group at high altitude. The probiotic group also exhibited a lower AMS score and enhanced acclimatization relative to the placebo group at high altitude, evidenced by higher SpO_2_ and lower AMS scores in treatment versus placebo groups. These results suggest bacteriotherapy as a novel, non-invasive intervention for high-altitude acclimatization.

## Highlights

• Bacteriotherapy has the potential to improve oxygen saturation (SpO_2_)
• 17 participants underwent a randomized, doubled-blinded, placebo-controlled study
• The probiotic group had higher SpO_2_ during daytime and nighttime at 3,800 m
• The probiotic group had lower acute mountain sickness (AMS) scores at 3,800 m

## Graphical abstract

**Figure:**
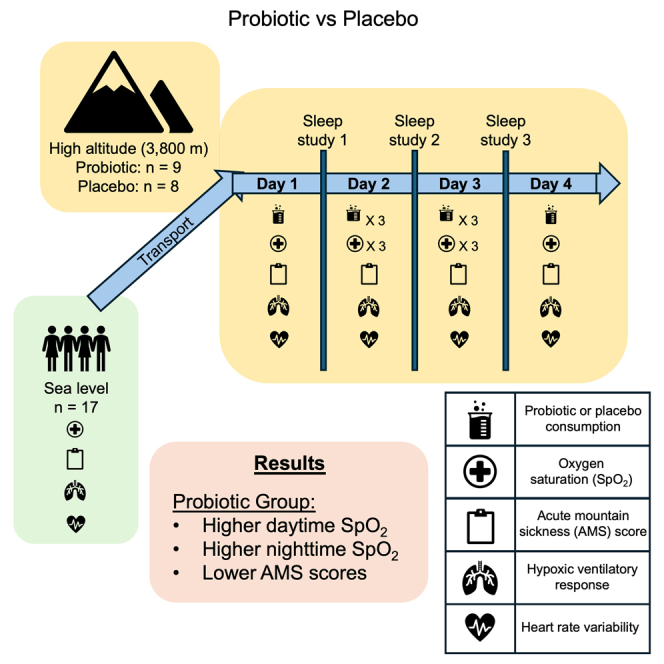

## Teaser

Human physiology; Environmental health

## Introduction

The reduced tension of oxygen (O_2_) at high altitude imposes various physiological stresses on humans, including decreased O_2_ saturation, disturbed sleep,^1^^,^^2^ increased sympathetic activation,^3^ reduced exercise capacity,^4^ and symptoms of acute mountain sickness (AMS), e.g., fatigue, headache, and shortness of breath.^5^^,^^6^ Despite these negative effects, millions of people ascend to high altitude every year for work and recreation,^7^ and many of these individuals become incapacitated or experience debilitating AMS symptoms.^6^ In extreme cases, some individuals develop life-threatening conditions, such as high-altitude pulmonary edema (HAPE) and high-altitude cerebral edema (HACE).^8^ Aside from slow ascent to altitude,^8^ limited options are available to improve acclimatization, require a prescription, and/or have side effects. Therefore, non-invasive interventions to aid in high-altitude acclimatization would be of great benefit.

The human body harbors internal and external microorganisms, and the gut microbiotia, which comprises bacteria, archaea, and eukaryotes in the gastrointestinal tract, plays a crucial role in maintaining health and contributing to disease.^9^ Given a large proportion of cardiac output is directed toward the intestines to transport O_2_ and other nutrients,^10^ modulation of the human-gut microbiota interface may be an avenue to improve systemic oxygenation. The probiotic examined in this study (Oxxyslab, formerly SLAB51) is a blend of eight different bacterial strains: *Streptococcus thermophilus*, two strains of *Bifidobacterium animalis* subsp. *lactis*, *Lactobacillus acidophilus*, *Lactobacillus helveticus*, *Lactobacillus paracasei*, *Lactobacillus plantarum*, and *Lactobacillus brevis*. Studies suggest that when bacteria from this probiotic interact with human intestinal epithelial cells, they increase stabilization of hypoxia inducible factor subunit 1 alpha (HIF-1α), a critical regulator of O_2_ homeostasis, potentially driving the intestine to utilize O_2_-independent pathways of metabolism and reducing O_2_ demand.^11^ This probiotic, has demonstrated promise in a wide range of health conditions such as Parkinson’s and Alzheimer’s diseases^12^^,^^13^^,^^14^^,^^15^^,^^16^, COVID-19,^17^^,^^18^^,^^19^ preterm birth,^20^ and oxidative stress from sleep loss, and could have the potential to improve acclimatization to high altitude.^18^^,^^20^

In this study, we investigate the effects of ingestion of this probiotic on acclimatization to high altitude in sea-level residents transported to 3,800 m (12,470 ft). Seventeen participants underwent a randomized, placebo-controlled, double-blinded interventional study: nine individuals ingested the probiotic throughout their stay at high altitude and eight individuals ingested a placebo. Participants underwent measurements at sea level and stayed up to four days and three nights at high altitude. O_2_ saturation, measured via pulse oximetry (SpO_2_), during both daytime and nighttime was the primary measurement for intervention efficacy, and secondary measurements included AMS score, apnea-hypopnea index (AHI), O_2_ desaturation index (ODI), hypoxic ventilatory response (HVR), hypercapnic ventilatory response (HCVR), hypercapnic HVR, hypoxic heart rate response (HHRR), resting heart rate (HR), blood pressure, heart rate variability (HRV), and fasting glucose levels. We hypothesized that individuals who received the probiotic would have a higher SpO_2_ during the daytime and nighttime compared to those who received the placebo, thereby improving high-altitude acclimatization. We collected additional secondary measurements of acclimatization to examine potential differences in physiological responses between the two treatment groups.

## Results

### Study participants

A total of 38 participants were assessed for eligibility for this study between June 29 and September 10, 2023. Of these 38 individuals, 14 were deemed ineligible, 24 were enrolled, seven decided to not complete the study, and 17 participated (full trial profile in Figure S1). The 17 participants (eight women and nine men) were split into three separate trips to high altitude between the dates of August 18 and October 8, 2024. Of these 17 participants, nine spent three nights and four days at high altitude, while eight spent only one night and two days at high altitude due to inclement weather that required evacuation. Details of the 17 participants’ baseline characteristics at sea level are listed in Table 1.

**Table 1:** Demographic and baseline measurements of study participants at sea level

Characteristic	Placebo, n = 8	Probiotic, n = 9
Sex		
Female	3 (38%)	5 (56%)
Male	5 (63%)	4 (44%)
Age (years)	22 ± 14	25 ± 14
BMI (kg/m^2^)	26.5 ± 5.3	25.1 ± 4.5
Sea-level SpO_2_ (%)	99.0 ± 1.5	98.0 ± 1.4
Sea-level resting HR (bpm)	60 ± 18	64 ± 10
Sea-level mean nighttime SpO_2_ (%)	96.0 ± 0.9	95.0 ± 1.1
Sea-level AHI 4%	1.4 ± 3.2	1.2 ± 5.8
Sea-level ODI 4%	1.7 ± 1.6	1.1 ± 5.5
Sea-level fasted glucose (mg/dL)	107 ± 10	106 ± 9
Sea-level systolic blood pressure (mmHg)	128 ± 14	114 ± 18
Sea-level diastolic blood pressure (mmHg)^a^	85 ± 11	70 ± 8
Sea-level HVR (Δ $\overset{\cdot }{\text{V}}$i/ΔSpO_2_)	0.32 ± 0.56	0.35 ± 0.32
Sea-level HCVR (Δ $\overset{\cdot }{\text{V}}$i/ΔETCO_2_)	0.53 ± 0.45	0.78 ± 0.34
Sea-level hypercapnic HVR (Δ $\overset{\cdot }{\text{V}}$i/(ΔETCO_2_ x ΔSpO_2_)	0.79 ± 0.86	1.03 ± 0.64
Sea-level HHRR (Δ HR/ΔSpO_2_)	0.89 ± 0.72	1.06 ± 0.58
Sea-level SDRR (ms)	66 ± 44	61 ± 29
Sea-level RMSSD (ms)	66 ± 81	61 ± 32
Sea-level LF Power (nu)	55 ± 10	58 ± 7
Sea-level HF Power (nu)	42 ± 16	39 ± 9
Sea-level LF/HF	1.33 ± 1.30	1.47 ± 0.80

a Significantly different between placebo and probiotic groups (*p* < 0.05). BMI, body mass index; SpO_2_, oxygen saturation measured by pulse oximetry; HR, heart rate; AMS, acute mountain sickness; AHI, apnea-hypopnea index; ODI, oxygen desaturation index; HVR, hypoxic ventilatory response; HCVR, hypercapnic ventilatory response; HHRR, hypoxic heart rate response; SDRR, standard deviation of the RR interval; RMSSD, root-mean-square of successive differences; LF, low-frequency; HF, high-frequency. Data are shown as mean ± SD.

### Oxygen saturation

**Figure 1:**
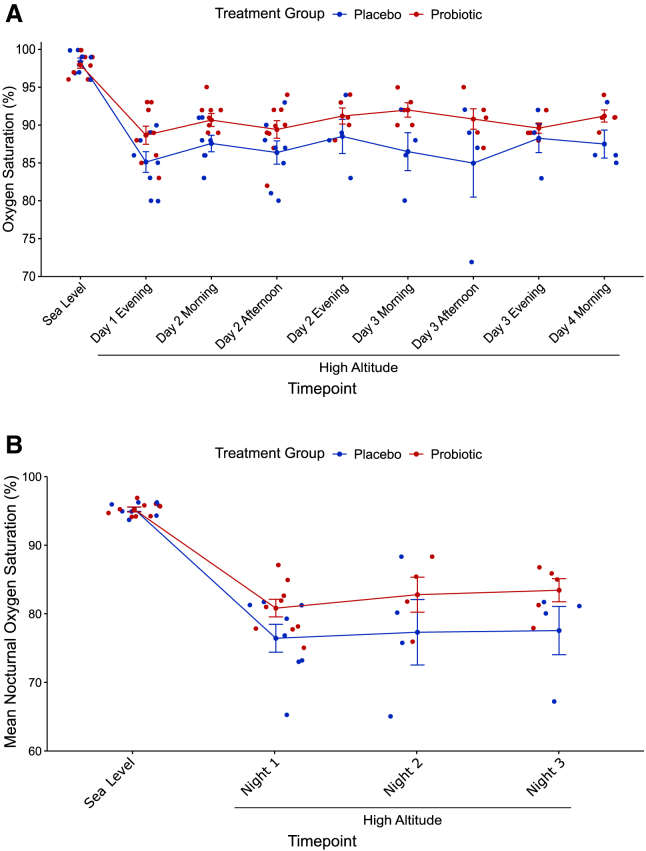
Individuals in the probiotic group had increased SpO_2_ at high altitude during both daytime and nighttime compared to the placebo group (A) All individuals experienced a decrease in SpO_2_ upon ascent to high altitude (*p* < 0.0001, paired Student’s t test). A two-way ANOVA revealed a significant effect of treatment group (*p* < 0.0001) but not time point (*p* = 0.47) on daytime SpO_2_ at high altitude, and a Bonferroni *post hoc* revealed a mean increase of 3.6% in the probiotic group. Error bars represent standard error (SE). (B) A two-way ANOVA also revealed a significant effect of treatment group (*p* = 0.011) but not time point (*p* = 0.66) on mean nocturnal SpO_2_ at high altitude, and a Bonferroni *post hoc* revealed a mean increase of 5.1% in the probiotic group. All 17 individuals had at least one night of sleep data at high altitude and nine individuals (five probiotic, four placebo) had three nights of sleep data at high altitude. Error bars represent SE.

All individuals had daytime SpO_2_ of at least 96% at sea level (Table 1; Figure 1A), and both groups, individuals who were later given the placebo (*n* = 8) or probiotic (*n* = 9), had a reduction in SpO_2_ upon ascent to high altitude (*p* < 0.0001 for both groups, paired Student’s t test). Compared to the placebo group (mean ± SD; 86.7 ± 4.4%), the probiotic group (90.2 ± 2.8%) had a higher daytime SpO_2_ at high altitude (*p* < 0.0001, two-way ANOVA with Bonferroni *post hoc*, effect size (ES) = 0.95, 95% confidence interval [CI] [2.1, 5.0], Figure 1A) with a mean increase of 3.6% but no effect of time point (*p* = 0.47, two-way ANOVA, Figure 1A). At sea level, all individuals had mean nocturnal SpO_2_ of at least 94% (Table 1). Compared to the placebo group (76.9 ± 6.6%), the probiotic group (81.9 ± 4.1%) had a higher mean nocturnal SpO_2_ at high altitude (*p* = 0.011, two-way ANOVA with Bonferroni *post hoc*, ES = 0.9, 95% CI [1.2, 9.0], Figure 1B) with a mean increase of 5.1% but no effect of time point (*p* = 0.66, two-way ANOVA, Figure 1B). A mixed-effects model predicting daytime SpO_2_ and considering other potential factors, such as HVR, age, sex, and body mass index (BMI) further supported the effect of increased SpO_2_ with probiotic ingestion compared to placebo (estimate = 2.5%, standard error = 1.1%, t-value = 2.31, Table S1). All mixed-effects models predicting mean nocturnal SpO_2_ and considering factors, such as HVR, HCVR, hypercapnic HVR, age, sex, and BMI were not significant (*p* > 0.05).

### Acute mountain sickness survey score

**Figure 2:**
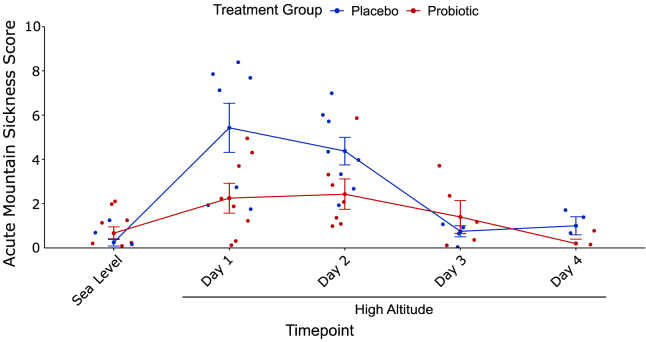
The probiotic group had decreased acute mountain sickness scores compared to the placebo group during the first two days at high altitude All individuals had an AMS score of two or less at sea level. The placebo group experienced a significant increase in AMS scores from sea level to the first day at high altitude (*p* = 0.0022, paired Student’s t test), while the probiotic group did not (*p* = 0.10, paired Student’s t test). The probiotic group had a lower AMS score during the first two days at high altitude compared to the placebo group (*p* = 0.0026, two-way ANOVA with Bonferroni *post hoc*) with a mean decrease of 2.5. Error bars represent SE.

At sea level, all individuals had an AMS score of two or less with most individual scores at zero (Table 1). The placebo group experienced a significant increase in AMS score from sea level to the first day at high altitude, while the probiotic group did not (*p* = 0.0022 and *p* = 0.10, paired Student’s t test, respectively). Compared to the placebo group (4.9 ± 2.4), the probiotic group (2.3 ± 1.8) had a lower AMS score during the first two days at high altitude (*p* = 0.0026, two-way ANOVA with Bonferroni *post hoc*, ES = 1.2, 95% CI [-4.1, −0.9], Figure 2) with a mean decrease of 2.5 but no effect of time point during the first two days at high altitude (*p* = 0.58, two-way ANOVA). The placebo group had a median AMS score of 7 (range: 2–8) on the first day at high altitude, and a median of 4 (range: 2–7) on the second day at high altitude. The probiotic group had a median of 2 (range: 0–5) on the first day at high altitude and a median of 2 (range: 1–6) on the second day at high altitude. Mean nocturnal SpO_2_ from the previous night at high altitude did not predict AMS score of the following day in a linear model that included treatment group, age, sex, and BMI for all three nights.

### Ventilatory and heart rate measurements during hypoxia, hypercapnia, and simultaneous hypoxia-hypercapnia

**Figure 3:**
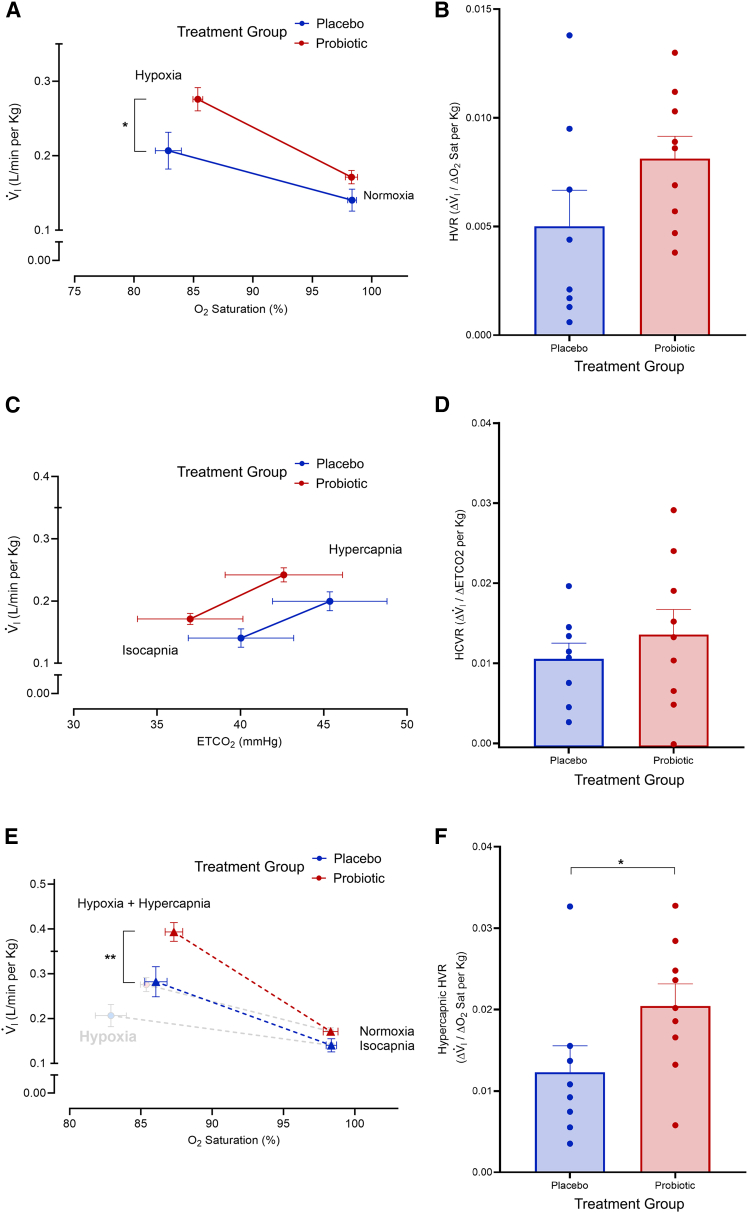
The probiotic group exhibited higher $\overset{\cdot }{\text{V}}$i during acute hypoxia exposure and higher hypercapnic HVRs at high altitude compared to the placebo group (A–D) The probiotic group (A) exhibited a higher $\overset{\cdot }{\text{V}}$i during acute hypoxia at high altitude compared to the placebo group (*p* = 0.012, unpaired t test) but (B) did not have a significantly higher HVR at high altitude (*p* = 0.062, unpaired t test). There were no significant differences between the treatment groups for (C) $\overset{\cdot }{\text{V}}$i during acute hypercapnia (*p* = 0.054, unpaired t test) and (D) HCVR at high altitude (*p* = 0.22, unpaired t test). (E) The probiotic group exhibited a higher $\overset{\cdot }{\text{V}}$i during combined hypercapnia-hypoxia (*p* = 0.002, unpaired t test) compared to the placebo group and both groups exhibited a higher $\overset{\cdot }{\text{V}}$i in combined hypercapnia-hypoxia compared to just hypoxia (depicted in faded images). (F) The probiotic group also exhibited a higher hypercapnic HVR at high altitude compared to the placebo group (*p* = 0.036, unpaired t test). One asterisk (∗) represents *p* < 0.05 and two asterisks (∗∗) represents *p* < 0.01. All error bars represent SE.

Ventilatory responses to hypoxia were measured at sea level and again on the second day (first full day) at high altitude in all participants. Both acute hypoxia and treatment group had a significant effect on $\overset{\cdot }{\text{V}}$i, and the $\overset{\cdot }{\text{V}}$i measured during acute hypoxia at high altitude was significantly higher in the probiotic (0.276 ± 0.047 L/min per kg) compared to the placebo group (0.207 ± 0.070 L/min per kg; *p* = 0.012, two-way ANOVA with Bonferroni *post hoc*, ES = 1.2, 95% CI [0.014, 0.124], Figure 3A). We did not find differences in $\overset{\cdot }{\text{V}}$i during exposure to normoxic gas (*p* = 0.40, two-way ANOVA with Bonferroni *post hoc*, Figure 3A). We further analyzed the slopes of these responses to determine the HVR (Δ $\overset{\cdot }{\text{V}}$i/ΔSpO_2_), which did not significantly differ between probiotic and placebo groups (*p* = 0.062, unpaired t test, Figure 3B).

We also quantified the effects of increased levels of CO_2_, and found that both acute hypercapnia and treatment group had a significant effect on $\overset{\cdot }{\text{V}}$i. However, the post-hoc analysis did not show significant differences during acute hypercapnia (0.241 ± 0.034 L/min per kg and 0.200 ± 0.043 L/min per kg for probiotic and placebo groups, respectively, *p* = 0.054, unpaired t test, Figure 3C) and no significant differences in HCVR (*p* = 0.22, unpaired t test, Figure 3D).

Exposure to hypoxic-hypercapnia produced an increased level of $\overset{\cdot }{\text{V}}$i compared to the values observed for acute hypoxia alone (values of $\overset{\cdot }{\text{V}}$i observed in acute hypoxia alone are included in faded circles for reference in Figure 3E). Both treatment group and simultaneous hypoxia-hypercapnia had significant effects on $\overset{\cdot }{\text{V}}$i. Our analysis revealed a significant increase of $\overset{\cdot }{\text{V}}$i during hypoxia-hypercapnia for participants who consumed probiotic (0.392 ± 0.064 L/min per kg) compared to placebo (0.282 ± 0.096 L/min per kg) at high altitude (*p* = 0.002, two-way ANOVA with Bonferroni *post hoc*, ES = 1.4, 95% CI [0.04, 0.18], Figure 3E). Participants who received the probiotic had a greater hypercapnic HVR (0.020 ± 0.008 Δ $\overset{\cdot }{\text{V}}$i/ΔSpO_2_ per kg) compared to placebo (0.012 ± 0.009 Δ $\overset{\cdot }{\text{V}}$i/ΔSpO_2_ per kg, *p* = 0.036, unpaired t test, Figure 3F).

Similar analyses of the HR response during hypoxia, hypercapnia, and combined hypoxia-hypercapnia showed that the HR response did not differ by treatment group (Figures S2A–S2C).

### Sleep measurements

**Figure 4:**
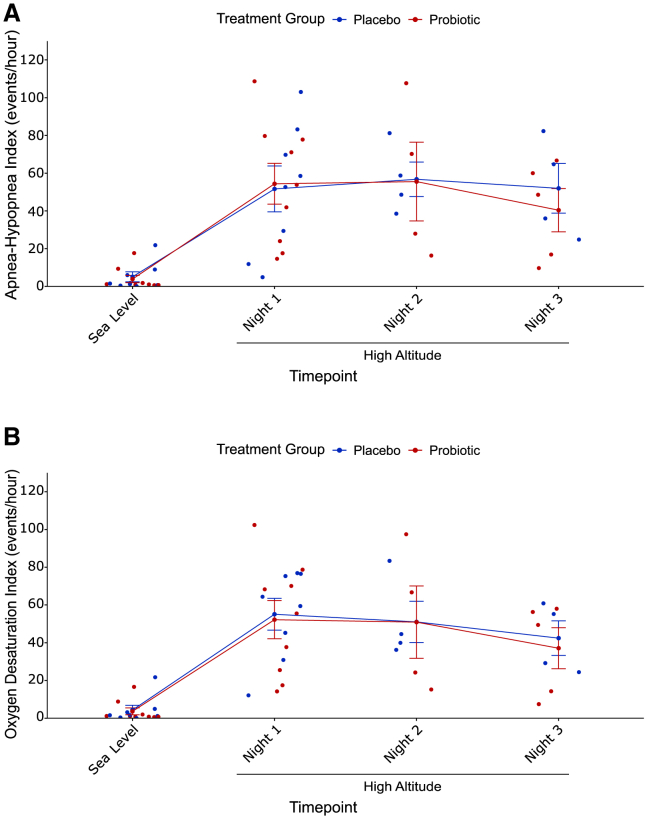
Treatment groups did not exhibit any differences in AHI or ODI at high altitude Both treatment groups experienced an increase in (A) AHI (*p* = 0.0078 for placebo group, *p* = 0.0012 for probiotic group, paired Student’s t tests) and (B) ODI (*p* = 0.00096 for placebo group, *p* = 0.00089 for probiotic group, paired Student’s t tests. There was no difference in (A) AHI or (B) ODI between the two groups at high altitude. Error bars represent SE.

At sea level, individuals who were in the placebo and probiotic groups did not exhibit differences in the AHI (*p* = 0.72, Student’s t test, Table 1) and the ODI (*p* = 0.83, Student’s t test, Table 1). The cohort of 17 individuals experienced an increase in AHI upon ascent to high altitude after ingesting the probiotic or placebo (*p* = 0.0078 for placebo group, *p* = 0.0012 for probiotic group, paired Student’s t tests, Figure 4A) as well as ODI (*p* = 0.00096 for placebo group, *p* = 0.00089 for probiotic group, paired student’s t tests, Figure 4B). However, there was no difference in AHI (*p* = 0.83, two-way ANOVA, Figure 4A), and ODI (*p* = 0.73, two-way ANOVA, Figure 4B) between the two groups. Neither AHI nor ODI from the first night at high altitude predicted AMS score of the following day in a linear model that included treatment group, age, sex, and BMI.

### Basic cardiovascular measurements

**Figure 5:**
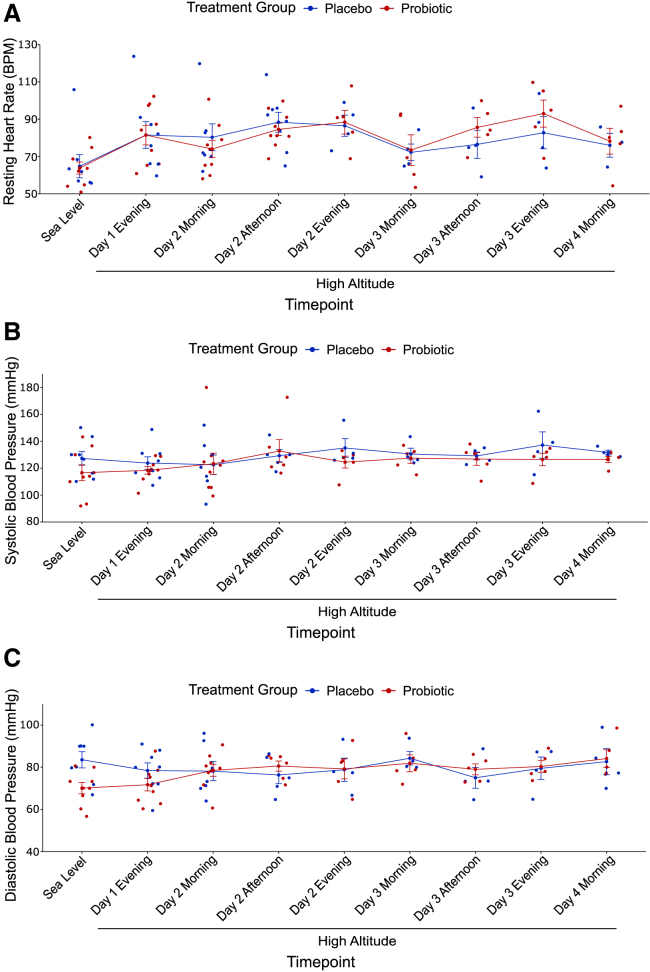
Treatment groups did not exhibit any differences in resting heart rate, systolic blood pressure, nor diastolic blood pressure at high altitude (A) Both groups experienced an increase in resting HR from sea level to high altitude (*p* = 0.0084 for placebo group, *p* = 0.0064 for probiotic group, Student’s t test) but did not differ in resting HR at high altitude. Error bars represent SE. (B) There was no difference in systolic blood pressure from sea level to high altitude in either group and no difference in systolic blood pressure between the two groups at high altitude. (C) Diastolic blood pressure decreased with exposure to high altitude in the placebo group (*p* = 0.045, Student’s paired t test) but did not change in the probiotic group. There was no difference in diastolic blood pressure between treatment groups at high altitude. Error bars represent SE.

Prior to administration of the probiotic or placebo, resting HR increased in all individuals from sea level to high altitude (*p* = 0.0084 for placebo group, *p* = 0.0064 for probiotic group, Student’s t test, Figure 5A) and did not differ between placebo and probiotic group at both sea level (Table 1) and at high altitude (*p* = 0.91, two-way ANOVA, Figure 5A). Systolic blood pressure did not differ between the two groups at sea level (*p* = 0.19, Student’s t test, Table 1) and did not change with exposure to high altitude for either group (*p* = 0.36 for placebo group, *p* = 0.69 for probiotic group, paired Student’s t tests, Figure 5B). There was no difference in systolic blood pressure between the two groups at high altitude (*p* = 0.22, two-way ANOVA, Figure 5B). Diastolic blood pressure was higher in the placebo group (83.5 ± 10.9 mmHg) compared to the probiotic group (70.1 ± 8.05 mmHg) at sea level (*p* = 0.014, Student’s t test, ES = 1.40, 95% CI [3.24, 23.6], Figure 5C) and decreased with exposure to high altitude in the placebo group (78.4 ± 10.3 mmHg, *p* = 0.045, Student’s paired t test, ES = 0.48, 95% CI [0.149, 10.1], Figure 5C) but not the probiotic group (*p* = 0.57, Student’s paired t test, Figure 5C). There was no difference in diastolic blood pressure between the two groups at high altitude (*p* = 0.92, two-way ANOVA, Figure 5C).

### Heart rate variability

**Figure 6:**
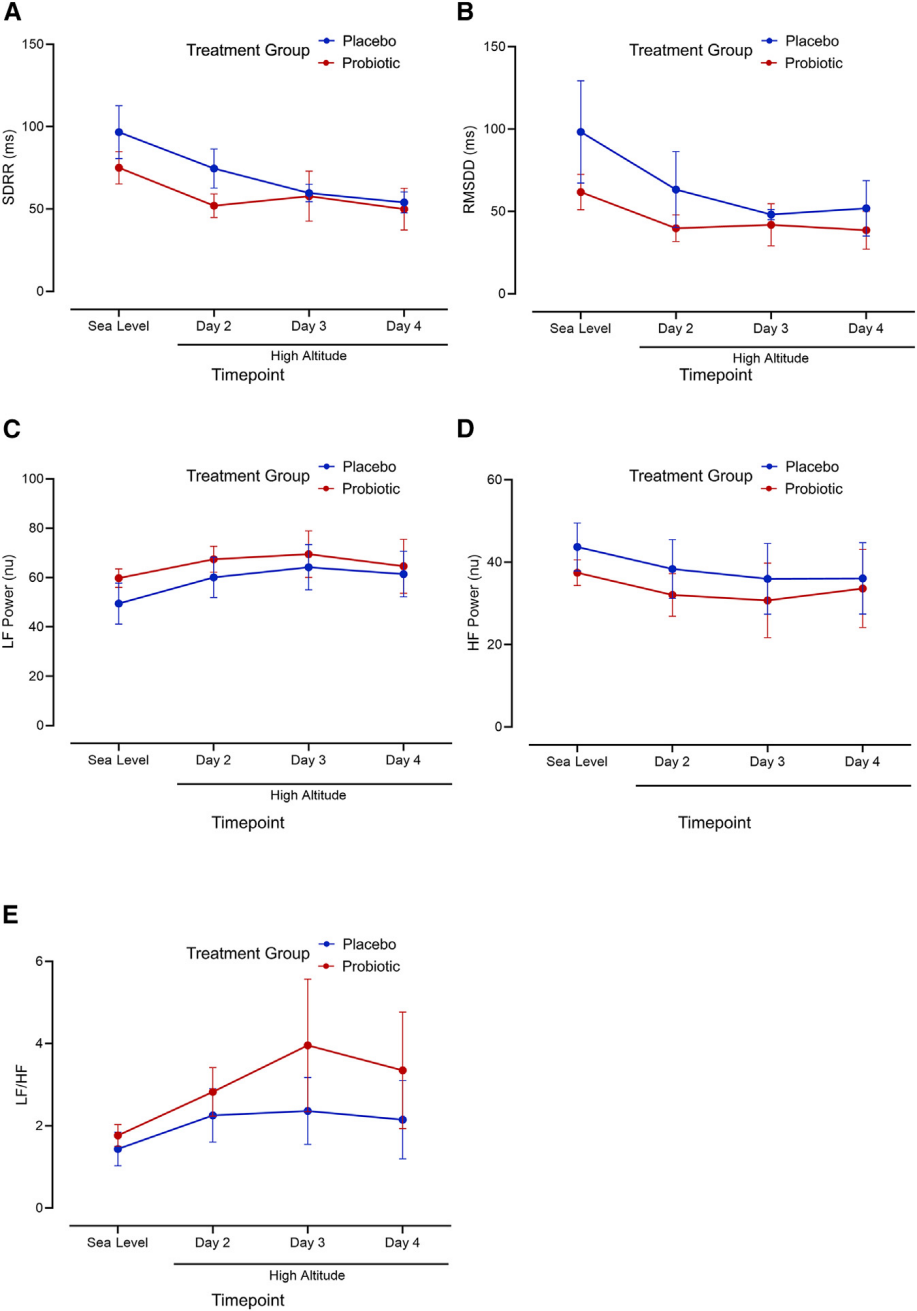
Treatment groups did not exhibit differences in measures of heart rate variability during exposure to high altitude (A) Both groups experienced a decrease in SDRR during the second day at high altitude compared to sea level (*p* = 0.028, Student’s t test) but no differences between treatment groups (*p* = 0.21, two-way ANOVA). (B) Neither group exhibited a change in RMSDD with exposure to high altitude (*p* = 0.074, Student’s t test) nor was there a difference between treatment groups in RMSDD (*p* = 0.10, two-way ANOVA). (C and D) There were no differences in LF or HF between sea level and the second day at high altitude in both groups (*p* = 0.081 and *p* = 0.155, respectively, Student’s t test) and no differences between groups at high altitude for both LF (*p* = 0.20, two-way ANOVA) and HF (*p* = 0.20, two-way ANOVA). (E) Both groups experienced an increase in LF/HR ratio during the second day at high altitude compared to sea level (*p* = 0.03, Student’s t test) but no differences between treatment groups (*p* = 0.12, two-way ANOVA). All error bars represent SE.

We completed analysis of HRV to obtain time-domain standard deviation of R-R intervals (SDRR) and the root mean square value of successive differences between normal heart beats (RMSDD) parameters, as well as frequency-domain low frequency (LF), high frequency (HF), and LF/HF values. In the entire group of participants, SDRR was significantly lower during the first morning at high altitude (62.0 ± 27.9 ms) compared to sea level (84.6 ± 36.1 ms, *p* = 0.028, Student’s t test, Figure 6A), and RMSDD did not differ between sea level and the first morning at high altitude (*p* = 0.074, Student’s t test, Figure 6B). Although there were no significant effects of altitude in the full cohort for LF (*p* = 0.081, Student’s t test, Figure 6C) or HF (*p* = 0.155, Student’s t test, Figure 6D), there was a significant increase in LF/HF at high altitude (2.58 ± 1.72) compared to the values obtained at sea level (1.62 ± 0.92, *p* = 0.03, Student’s t test, Figure 6E). There were no significant differences in LF/HF between treatment groups at high altitude (*p* = 0.12, two-way ANOVA, Figure 6E), time-domain parameters of SDRR and RMSDD (*p* = 0.21 and 0.10 respectively, two-way ANOVA, Figures 6A and 6B), and frequency-domain parameters of LF (*p* = 0.20, two-way ANOVA, Figure 6C) and HF (*p* = 0.196, two-way ANOVA, Figure 6D).

### Fasting glucose

**Figure 7:**
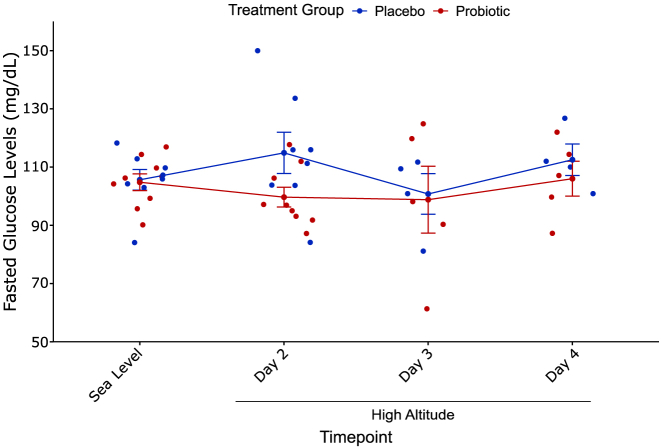
Treatment groups did not exhibit differences in fasted glucose at high altitude Both treatment groups did not experience a significant change in fasted glucose from sea level to the first morning (Day 2) at high altitude and did not differ in fasted glucose levels at high altitude. Error bars represent SE.

Fasting glucose did not significantly change from sea level (Table 1) to high altitude in either group but did, on average, increase in the placebo group by a mean of 9.25 mg/dL (*p* = 0.20, Student’s t test, Figure 7) and decrease in the probiotic group by a mean of 5.11 mg/dL (*p* = 0.16, Student’s t test, Figure 7). Fasting glucose did not differ between the placebo and probiotic groups at high altitude (*p* = 0.091, two-way ANOVA, Figure 7).

## Discussion

This study provides the first evidence that ingestion of this probiotic has the capability to improve acclimatization to high altitude in humans. We explored its impact on high-altitude acclimatization in sea-level residents in a randomized, placebo-controlled, double-blinded interventional study. The probiotic had never been tested in this context before. Our findings show that probiotic ingestion increases daytime and nighttime O_2_ saturation, decreases symptoms of AMS, and increases ventilation at high altitude. We also measured AHI, ODI, resting HR, blood pressure, HRV, and fasting glucose, all of which did not differ significantly between the two treatment groups.

The probiotic group had higher daytime SpO_2_ than the placebo group at high altitude with a mean increase of 3.6% (Figure 1A). When predicting daytime SpO_2_ in a mixed-effects model considering other factors such as ventilation, age, sex, and BMI, the effect size of the probiotic was 2.5%, with the model indicating that HVR at high altitude is also a significant contributor to daytime SpO_2_ (Table S1). While the HVR is known to minimize the reduction in SpO_2_ one would otherwise experience due to hypobaric hypoxia,^22^ the mixed-effects model indicates that this specific probiotic also has an independent contributing effect on SpO_2_. It is plausible alterations in small molecule and/or gaseous signals result in altered, physiological effects.^23^^,^^24^ In any case, the mechanism of action quickly affects O_2_ saturation as SpO_2_ measurements were taken approximately two to three hours after the first ingestion at high altitude. These values are greater than the increase in SpO_2_ seen with acetazolamide when ascending to high altitude in studies conducted at 4,937 m and 3,658 m, which report a 1.8%^25^ and 2.5%^26^ improvement in SpO_2_, respectively. However, these values are lower than the increase in SpO_2_ from acetazolamide seen in an exercise study at 3,459 m where Bradwell et al.^27^ reported a mean increase of 6.3% and an exercise study at a simulated altitude of 3,500 m where Bradbury^28^ reported a mean increase of 5.0%. In all of these studies it is important to note that the ascent profiles varied compared to ours.

The probiotic group also had a higher nighttime SpO_2_ than the placebo group at high altitude with an even higher mean increase than the daytime SpO_2_ of 5.1% (Figure 1B). This value is similar to the increase in nocturnal SpO_2_ seen with acetazolamide in a meta-analysis that reported a mean increase of 4.75% in nocturnal SpO_2_.^29^ At high altitude, humans experience a reduction in SpO_2_ during sleep due to decreased ventilatory drive^30^^,^^31^ and many experience sleep-disordered breathing (SBD) that further depresses nighttime SpO_2_.^2^^,^^32^ These depressions in SpO_2_ during sleep can result in a sequelae of negative physiological health impacts, such as inflammation, increased sympathetic activation, oxidative stress, and increased risk of cardiovascular disease.^33^^,^^34^^,^^35^^,^^36^^,^^37^^,^^38^ A previous study indicated that this probiotic may reduce inflammation and oxidative stress caused by chronic sleep restriction in mice,^21^ and this study indicates it may also attenuate the reduction in nighttime SpO_2_ experienced by humans during sleep at high altitude, though with minimal effects on SDB itself (discussed further in the following text). Additionally, a previous study has indicated that certain bacteria may alleviate acute intestinal injury due to hypoxia in mice.^39^

Compared to the placebo group, the probiotic group had a lower AMS score during the first two days at high altitude (Figure 2). The Lake Louise AMS survey asks participants to assess severity of their symptoms on a numerical scale^40^ and has been utilized by a wide range of studies to assess AMS symptom severity.^40^^,^^41^^,^^42^^,^^43^ Many humans experience AMS symptoms when they ascend to high altitudes, and the severity of the symptoms are usually highest on the first and second days at elevation.^5^^,^^6^ This study indicates that consumption of the probiotic may reduce the severity of AMS symptoms as evidenced by lower AMS scores in the probiotic group when compared to the placebo group. How exactly the probiotic may alleviate AMS symptoms is unknown, though a higher SpO_2_ is known to be associated with a lower AMS score.^42^ Within our cohort, mean nocturnal SpO_2_, AHI, or ODI did not predict AMS score the following day in a mixed-effects model that included treatment group, age, sex, and BMI. Previous studies indicated that sleep disturbance at high altitude did not correlate with AMS severity the following day,^44^^,^^45^ which prompted a change in the Lake Louise scoring of AMS severity and removed sleep as part of the questionnaire in 2018.^40^ Although limited to measurements of mean nocturnal SpO_2_, AHI, and ODI, which may not capture pivotal patterns of hypoxemia during sleep,^46^^,^^47^ our results support this notion based on the measurements obtained.

The probiotic group had a higher $\overset{\cdot }{\text{V}}$i during the hypoxic stage of the ventilation protocol than the placebo group (Figure 3A), but the overall HVRs were not significantly different between the two groups (Figure 3B). Ventilation increases with exposure to hypoxia,^48^^,^^49^ and this increase in ventilation is a strong indicator of acclimatization to the hypoxia experienced at high altitude.^50^ Considering ventilation is a key contributor to SpO_2_,^48^^,^^51^ we quantified ventilation in room air, normoxia, and hypoxia at high altitude. Our mixed-effects model predicting daytime SpO_2_ indicates that HVR at high altitude did indeed significantly predict daytime SpO_2_ (Table S1), though this was found to be independent of treatment group. A higher $\overset{\cdot }{\text{V}}$i during the hypoxic stage in the probiotic group at high altitude compared to the placebo group indicates probiotic consumption may increase ventilation, but this difference did not extend to the HVRs of the respective groups. The HVR is highly variable among individuals,^22^ and a larger sample size may be needed to detect a significant difference between treatment groups. It is also feasible that there may be interactions among the immune system, ventilation, and probiotic consumption. Basaran et al.^52^ found that ibuprofen decreased the HVR in humans after 48 hours at high altitude. This result was previously investigated in rats, where administration of ibuprofen prevented the hypoxia-dependent increase of ventilation and levels of inflammatory makers in the nucleus tractus solitarii (involved in control of breathing).^53^ Consumption of bacteria, which in this case are all gram-positive, may elicit an immune response, though more studies must be conducted to determine if this occurs and if these factors may contribute to the fast-acting effect that probiotic administration had on SpO_2_.

An analysis of HCVR at sea level and high altitude showed no significant differences in HCVR between treatment groups at high altitude (Figure 3D). HCVR is believed to play a role in SDB susceptibility at high altitude. Increased sensitivity to CO_2_ during sleep may drive increased respiration and exhalation of CO_2_, leading to central sleep apneas, though this potential connection requires further study.^2^^,^^54^ A previous study provides evidence that manipulation of the gut microbiota affects the HCVR in rats,^55^ warranting further investigations into a potential connection between the gut microbiota and ventilatory responses via the gut-brain-lung axis. An analysis of combined hypoxia and hypercapnia on ventilation, also known as the hypercapnic HVR, revealed a significantly higher hypercapnic HVR in the probiotic group compared to the placebo group at high altitude (Figure 3F). The combination of hypoxia and hypercapnia produces a synergistic effect^49^; therefore, while hypoxic and hypercapnic signals alone were close to significant, discernible differences in ventilatory responses between treatment groups was detected during the hypoxic hypercapnic stage of the protocol. An analysis of the HR response during the HVR, HCVR, and hypercapnic HVR revealed no differences between treatment groups (Figure S2). In combination, these results suggest the probiotic may augment ventilatory, but not cardiac, responses to O_2_ and CO_2_, which warrant further investigation.

Although both the placebo and probiotic groups experienced an increase in AHI and ODI from sea level to high altitude (Figures 4A and 4B, respectively), there was no difference between the two groups at high altitude for both measurements (Figures 4A and 4B). These results suggest that the differences observed for nocturnal SpO_2_ between the two groups were not primarily driven by these metrics of SDB, and that the probiotic may not alleviate these patterns of intermittent hypoxia experienced with SDB at high altitude. When humans ascend to high altitude, they often experience intermittent hypoxia during sleep in addition to sustained hypoxia,^2^^,^^56^ and this combination may lead to poor cardio-metabolic outcomes.^56^ Therefore, while the probiotic may not ameliorate SDB and intermittent hypoxia exposure, it may mitigate the extent of hypoxia experienced at high altitude, thereby still having a positive impact on cardio-metabolic health.

Resting HR of both groups increased with exposure to high altitude but did not differ between the two groups at high altitude (Figure 5A). This increase in resting HR from sea level to high altitude has been previously observed.^57^^,^^58^^,^^59^ Systolic blood pressure did not change with high-altitude exposure, which contrasts with other studies that exposed sea-level residents to high altitude,^57^^,^^58^ though an earlier study noted no change in blood pressure with high-altitude exposure in sea-level residents,^59^ and there was no difference between the placebo and probiotic groups (Figure 5B). Diastolic blood pressure was higher in the placebo group at sea level and decreased with high-altitude exposure in the placebo group but did not change in the probiotic group, resulting in no overall difference in diastolic blood pressure at high altitude between the two groups (Figure 5C), and this initial difference in diastolic blood pressure at sea level may be due to chance.

Exposure to high altitude induced changes in HRV, a measurement that approximates sympathetic autonomic nervous system activation,^60^ indicated by lower measures of SDRR and higher levels of LF/HF compared to sea-level values (Figures 6A and 6E). However, we did not observe any differences in HRV between treatment groups (Figure 6). Previous studies indicate that hypoxia increases sympathetic activation as showcased by measures of HRV at high altitude^61^^,^^62^ and in hypobaric chambers^63^^,^^64^ as well as with direct measurements of muscle sympathetic nerve activity in lowlanders traveling to high altitude and populations with a history of residence at high altitude.^3^^,^^65^^,^^66^ Several previous studies have examined the effects of probiotic usage on HRV but none used the particular probiotic from this study. Kikuchi-Hayakawa et al.^67^ found that probiotic containing *Lacticaseibacillus paracasei* strain Shirota decreased values of LF/HF measured during the afternoon in office workers, and Quero-Calero et al.^68^ found no effects of probiotic usage on HRV in athletes and people with sedentary habits. Another study found a positive association between *Lachnospiraceae incertae sedis* counts and higher SDRR in a cohort of 950 Japanese individuals.^69^ While we controlled for known circadian rhythm effects on HRV^70^^,^^71^^,^^72^ by only performing our HRV measurements in fasted conditions in the morning, it is unclear how much existing bacterial compositions may have contributed to the variability seen in HRV measurements within our cohort.

Fasting glucose did not increase in either group with high-altitude exposure (Figure 7) in contrast with previous studies that exposed sea-level residents to high altitude.^73^^,^^74^ Interestingly, the placebo group exhibited a trend toward an increase in fasting glucose levels with high-altitude exposure while the probiotic group exhibited a decreased trend. This observation, in conjunction with the observed trend toward a lower fasting glucose level in the probiotic group on the second day at high altitude (Figure 7), suggests that the probiotic may improve glucose intolerance at high altitude. This suggestion is further supported by a previous study that showed improvement in glucose uptake in Alzheimer’s disease mouse models with probiotic treatment.^14^

How this probiotic improves O_2_ saturation remains to be clarified, although it appears to exert effects across multiple levels. Although some evidence suggests modulation of the ventilatory responses to hypoxia (Figure 3), the mixed-effects model predicting daytime SpO_2_ indicates that the probiotic also has a positive effect on SpO_2_ that is separate from the HVR. A previous study suggests ingestion of this probiotic may stabilize HIF-1α and attenuate inflammatory responses induced by exposure to lipopolysaccharide in human intestinal epithelial cells.^11^ Another potential mechanism may involve mediation through short-chain fatty acids (SCFAs). SCFAs, while typically associated with distal microbiota and bacterial colonization not involved in the fast-acting effects described, are known to facilitate communication between microbial and host cells, including neurons.^24^ Butyrate is an SCFA utilized by human intestinal epithelial cells as an energy source and also directly inhibits prolyl hydroxylases (PHDs), in turn helping to stabilize HIF1A.^75^ It may be feasible that probiotic ingestion may interact with the proximal intestine and increase SCFA and/or other small molecule secretions, such as butyrate, thereby having a stabilizing effect on HIF. Additionally, gaseous transmitters linked to the microbiota, such as carbon monoxide (CO) and hydrogen sulfide (H_2_S) with further implications for nitric oxide (NO),^24^ are associated with control of breathing metrics in animal models,^76^ which we hypothesize influence patterns of breathing during the daytime and also during sleep in humans. Further studies investigating the mechanism of this probiotic are needed.

Current methods to improve acclimatization to high altitude include slow and steady ascent to altitude^6^ and/or use of acetazolamide.^8^^,^^77^ This study provides evidence for an alternative, non-invasive intervention to improve acclimatization to high altitude within a relatively short period of time by ingestion of a specific probiotic. Furthermore, this study suggests that targeted bacteriotherapy may be a previously underutilized and overlooked method of improving O_2_ saturation. This application is relevant in the context provided in this study (healthy sea-level residents ascending to higher elevations, such as for recreational or employment reasons) but may also be expanded to other contexts, such as individuals with diseases related to O_2_ homeostasis, high-altitude residents, and military personnel operating in hypoxic environments. Individuals with diseases, such as chronic obstructive pulmonary disease,^78^ sickle cell disease,^79^ and sleep apnea,^35^ among others, struggle with O_2_ homeostasis—improvement of O_2_ saturation by bacteriotherapy may be a method to increase baseline SpO_2_ during both daytime and nighttime. Millions of individuals already reside at high altitude^80^ and, in a warming world, more may seek out higher altitudes as relief from climate change. As non-adapted individuals, they may benefit from interventions that improve O_2_ saturation. Even populations with high-altitude ancestry for hundreds of generations suffer from maladies, such as chronic mountain sickness, related to high altitude in their local environments.^81^ Acetazolamide has been previously tested for its efficacy in combatting chronic mountain sickness,^82^^,^^83^ and bacteriotherapy may be a new tool to add to the toolkit. Lastly, military operations can occur in high-altitude regions where altitude sickness can be a serious detriment,^84^ and military aviators constantly face the threat of hypoxia.^85^ Therefore, targeted bacteriotherapy provides a novel avenue to help combat the threat of hypoxia in various environments and potential pathological conditions.

### Limitations of the study

While this study is the first to investigate this probiotic in humans acclimatizing to high altitude, it comes with limitations. This study only investigated one dosage amount, and future studies would benefit from a dose-response experiment to determine effective bacterial loads. Additionally, we observed significant physiological differences between the two groups. Given the fast-acting nature of the probiotic, future studies should aim to investigate metabolomic, proteomic, and other biomarker signals which may provide mechanistic insight into the increase of SpO_2_ with probiotic ingestion. Long-term studies are further warranted to quantify alterations in the microbiome itself via fecal sample analyses. Additionally, transit time from ingestion to the gut varies by individual, and transit time in the stomach can range from five minutes to two hours.^86^^,^^87^ A previous study with this probiotic found effects on SpO_2_ within preterm infants within two hours,^20^ which is slightly longer than the 90 minutes after first ingestion within this study. Furthermore, our sample size of 17 individuals could be expanded in future studies to determine whether significant difference between the two treatment groups for our main outcome SpO_2_ during both daytime and nighttime is replicated. Additionally, while our cohort contained only Latina/Latino individuals, future studies could be expanded to include other groups to determine if these results are replicable across different ancestral and/or ethnic backgrounds.

## Resource availability

### Lead contact

Further information and requests for resources and experimental design should be directed to and will be fulfilled by the lead contact, Tatum Simonson (tsimonson@health.ucsd.edu).

### Materials availability

This study did not generate new unique reagents.

### Data and code availability

All data reported in this paper that is not considered protected participant data will be shared by the lead contact upon request. All original code is available from the lead contact upon request. Any additional information required to reanalyze the data reported in this paper is available from the lead contact upon request.

## Acknowledgments

We sincerely thank all participants for their time and contributions to this study. This study was primarily funded by the LEA Altitude Performance Fund, which provided the probiotic and placebo treatment for the study, but had no role in study design, data collection, data analysis, data interpretation, or writing of the report. We further acknowledge support of this work by the Wu Tsai Human Performance Alliance and the Joe and Clara Tsai Foundation. Other funding sources include the White Mountain Research Center Minigrant awarded to J.J.Y., the 10.13039/100000002National Institutes of Health R01HL145470 awarded to T.S.S., and the 10.13039/100000097National Center for Research Resources UL1TR001442 awarded to A.M. The authors thank the staff of the White Mountain Research Center for their support at Barcroft Station. We thank Frank Powell for providing expertise in experimental design and results interpretation and Janelle Fine for overseeing the blinding of the probiotic and placebo and assisting with hardware issues encountered throughout the study.

## Author contributions

J.J.Y., experimental design, acquisition of data, processing of data, data and statistical analysis and interpretation of data, and drafting of the original manuscript; E.A.M., experimental design, acquisition of data. processing of data, data and statistical analysis and interpretation of data, and drafting of the original manuscript; H.C., acquisition of data and processing of data; K.K., acquisition of data and processing of data; T.O., acquisition of data and processing of data; S.F., acquisition of data; E.G., acquisition of sleep data and processing of sleep data; A.S., acquisition of sleep data and processing of sleep data; P.D., processing of sleep data; E.V.Y., acquisition of data; L.A.B., acquisition of data; A.L., acquisition of data, A.S.A., acquisition of data; J.E.O., medical oversight of study and interpretation of data; E.C.H., acquisition of data; A.M., oversight of sleep data collection; T.S.S., experimental design, acquisition of data, interpretation of data, and drafting and revision of the original manuscript.

## Declaration of interests

T.S.S. and A.M. are funded by the National Institutes of Health. A.M. reports income from Eli Lilly, Zoll, Powell Mansfield, and Livanova. ResMed provided a philanthropic donation to UC San Diego.

## STAR★Methods

### Key resources table

**Table:** 

REAGENT or RESOURCE	SOURCE	IDENTIFIER
**Software and algorithms**		

RStudio	RStudio	Version 2023.6.0.421
randomizeR	randomizeR	Version 3.0.0
XLSTAT	XLSTAT	Version 19.2.2
lme4	lme4	Version 1.1.35.1
DHARMa	DHARMa	R package version 0.4.6

### Experimental model and study participant details

We conducted a randomized, double-blinded, placebo-controlled study including 17 participants (eight women and nine men) to test the effects of a probiotic on acclimatization to high altitude. We collected measurements from participants at sea level in a human laboratory space at the University of California, San Diego (UC San Diego) and at high altitude at the University of California White Mountain Research Center’s (WMRC) Barcroft Station (3,800 m) during a stay of up to four days (three nights). All non-sleep measurements were conducted in laboratory spaces at UC San Diego or at Barcroft Station. Sleep measurements at sea level were collected in participants’ homes and sleep measurements at high altitude were collected at Barcroft Station. Participants were transported via automobile during an approximately eight-hour drive from UC San Diego to Barcroft Station. Participants consumed either the probiotic or placebo upon arrival at Barcroft Station and up to three times a day during the duration of their stay. The study was approved by the UC San Diego Institutional Review Board (Protocol #807971) and registered on clinicaltrials.gov (Protocol ID NCT06552806).

We recruited self-identified Latino/Latina individuals, the second largest ethnic group in San Diego County, from the San Diego metropolitan and surrounding areas for this study. The response to high altitude is highly variable and partially driven by genetic background,^50^^,^^88^ so we chose to recruit from a single group to reduce variability in responses for this first study of the probiotic at high altitude. Participants were recruited via word-of-mouth, flyers posted throughout the greater San Diego metropolitan area, and emails sent to various Latin American associations and other community groups. Participants were considered eligible if they identified as being Latino/Latina, were at least 18 years old and less than 66 years old, and in good general health. Participants were deemed ineligible if they had a history of cardiovascular or pulmonary disease, currently smoked or used tobacco (more than one cigarette a day), had a body mass index (BMI) greater than 35, and/or an STOP-BANG score for obstructive sleep apnea equal or greater than three (https://www.mdcalc.com/calc/3992/stop-bang-score-obstructive-sleep-apnea). Individuals were also excluded for any use of medications or agents that might interfere with physiological measurements, if they traveled above 2,500 m (8,202 feet) of elevation during the four weeks prior to the initial baseline measurements at sea level and throughout the duration of the study. Eligibility was first determined by an online eligibility form and confirmed after physiological measurements were taken in a UC San Diego laboratory space. Participants provided written informed consent prior to any measurements taken at UC San Diego.

### Method details

#### Randomization and masking

Prior to the high-altitude portion of the study, participants were assigned evenly to either Treatment Group A or B by the computer randomization program randomizeR (Version 3.0.0). Both researchers and study participants were blinded as to whether Group A or B received the probiotic or placebo. Each dose of the probiotic and placebo was contained in a non-transparent sachet and labeled A or B by an assistant who was not part of the study, and only this individual knew which group received which treatment. Only after all data had been collected and preliminary data analyses comparing measurements between the treatment groups were complete were the researchers unblinded.

#### Probiotic and placebo ingestion and administration

Each sachet, labeled A or B, contained six grams of powder mixture. The placebo consisted of all components present in the probiotic formulation except for the bacteria, which were replaced with additional maltose or other safe food additives. The sachets containing the probiotic formula consisted of 800 billion live bacteria. Each mixture was dissolved in 100 mL of water and mixed thoroughly. Participants ingested the mixture in front of a researcher who recorded each ingestion and noted the time of ingestion. Participants ingested the probiotic or placebo within 15 minutes after every meal–after dinner on the day of arrival to high altitude, after breakfast, lunch, and dinner on days two and three, and after breakfast on day four, for a total of up to eight doses. Participants were provided with the same foods at all meals to reduce variable effects of different diets. All probiotic and placebo sachets manufactured by EOS2021 Srl, Ardea (Roma, Italy) were stored in refrigerated conditions or in a cooler during transportation (2°–8°C) and away from light until consumption. While the product can remain up to one week out of the refrigerator up to a maximum of 25°C without affecting its quality (Prof. Claudio De Simone, personal communication), the probiotic and placebo sachets were never out of refrigerated conditions for more than 30 minutes.

#### Oxygen saturation and heart rate measurements

Daytime SpO_2_ and resting HR were measured by a pulse oximeter probe (Radical-7, Masimo Corporation, Irvine, CA) that was secured to the forehead by a headband. All measurements were taken at rest in a seated position. Measurements were collected up to three times a day: once in the evening after arrival at high altitude and at least 90 minutes after consumption of the first dose of probiotic or placebo, three times a day during the second and third day at high altitude (generally in the morning, afternoon, and evening), and once in the morning on the last day at high altitude. Nighttime SpO_2_ and digital blood flow were measured by fingertip peripheral arterial tonometry and pulse oximetry using WatchPAT device (Itamar Medical, Caesarea, Israel).

#### Acute mountain sickness score

Acute mountain sickness severity was assessed by the 2018 Lake Louise AMS survey^40^ to determine severity of participants’ AMS symptoms. Participants answered the four questions from the survey on an iPad at sea level during initial measurements and every day at high altitude. The first survey was taken at least 1 hour after the initial ingestion of probiotic or placebo and at least 2 hours after the initial ascent. The results of the four questions were added up to determine a total AMS score for each day.

#### Sleep measurements

All sleep data were recorded using a WatchPAT wrist-worn Home Sleep Apnea testing device, a device proven for detection of obstructive sleep apnea^89^ and central sleep apnea.^90^ The WatchPAT device is equipped with pulse oximetry, arterial tonometry, an actigraph, and an accelerometer. Respiratory events were measured using PAT signal attenuation, changes in heart rate, and O_2_ desaturation and were subsequently analyzed through the WatchPAT proprietary software algorithm.^91^ Total sleep time was derived from the actigraphy signal, and sleep stage was calculated through PAT analysis, the specifics of which have been previously mentioned.^92^ Device data were analyzed using automated scoring through the WatchPAT’s proprietary algorithm and subsequently underwent scoring by a registered polysomnographic technologist.^93^

#### Ventilatory and heart rate responses to hypoxia and hypercapnia

Participants completed a control of breathing protocol as used in our previous high-altitude studies.^94^ At sea level, participants completed an abbreviated, approximately 10-minute, version of the full control of breathing protocol, which provided an opportunity for them to familiarize with the experimental set up and to establish appropriate gas flows for targeted SpO_2_ and end-tidal partial pressure of carbon dioxide (PetCO_2_). Participants returned to complete the full protocol at sea level after a >30-minute rest period, which provided sufficient time for recovery from hypoxic ventilatory decline that could be induced by the abbreviated hypoxia exposure.^95^ At high altitude, participants completed the full protocol on the second day after spending one complete night at high altitude.

During testing, participants sat in a chair and a mask was placed over the mouth and nose (7600 V2 Oro-Nasal Mask, Hans Rudolph, Shawnee, KS). Leaks were checked by asking the participant to inhale against a closed inspiratory valve to ensure a vacuum was produced. The mask was connected to a one-way vented breathing circuit with a non-rebreathing valve (2700 Series, Large, Hans Rudolph). A three-way valve upstream of the mask allowed either room air or gas mixtures of nitrogen, O_2_, and CO_2_ to flow into the circuit. O_2_ and CO_2_ were continuously sampled right before the mask or non-rebreathing valve directly in front of the mouth, respectively (Model 17620 and 17515A, VacuMed, Ventura, CA, USA). Inspiratory flow was measured by a pneumotachograph (Fleisch No. 3, OEM Medical Inc., Richmond, VA, USA) upstream of the mask and connected to a differential pressure transducer (model CD15, Validyne, Northridge, CA, USA). SpO_2_ and HR were measured by a pulse oximeter (Radical-7, Masimo Corporation Irvine, CA, USA) with a surface probe secured on the forehead. All analog signals were processed through a PowerLab 8/30 (ADInstruments, Colorado Springs, CO, USA) and sent digitally to a laptop computer. Raw data were recorded in LabChart 8 (ADInstruments). Gas mixtures were manually controlled with a three-channel rotameter flow meter (Matheson Gas Products, Montgomeryville, PA, USA) that delivered mixtures upstream of the three-way valve at flow rates sufficient to prevent rebreathing.

We continuously measured SpO_2_, fraction of inspired O_2_ (FI_O2_), fraction of inspired CO_2_ (Fi_CO2_), tidal volume (V_T_), respiratory rate, minute ventilation ($\overset{\cdot }{\text{V}}$i), PetCO_2_, and HR during the protocol. Participants breathed ambient air for three to five minutes followed by 10 minutes of mild hyperoxia (PO_2_∼230 mmHg, simulating 30% FI_O2_ at sea level). This hyperoxic phase was required to reverse potential hypoxic ventilatory decline in participants, as applied in previous studies with people exposed to continuous hypoxemia at high altitude.^48^

To determine the HVR in isocapnic conditions, participants breathed a normoxic gas mixture during five minutes (PO_2_∼159 mmHg, simulating a level equivalent of 21% FI_O2_ at sea level), followed by five minutes of isocapnic hypoxia as we targeted three minutes of stable SpO_2_ that produced a decrease of at least 10% from the levels recorded during previous hyperoxic measurements.^94^ Isocapnia was achieved throughout the hypoxic phase by manually adding CO_2_ to maintain the average PetCO_2_ value observed during the last minute of the previous hyperoxic period. Isocapnic hypoxic events with PetCO_2_ more than 2 mmHg different than the PetCO_2_ observed during the previous normoxic condition within the same participant were removed. Participants were then exposed to five minutes of normoxic hypercapnia by bringing the levels of O_2_ to the equivalent of 21% FI_O2_ at sea level with a 5 mmHg increase in PetCO_2_ above the value observed during normoxic isocapnia. The HVR and hypercapnic HVR were calculated as the change in ventilation per decrease in SpO_2_ (Δ $\overset{\cdot }{\text{V}}$i/ΔSpO_2_) under each condition of isocapnic hypoxia and hypercapnia, respectively. The normoxic hypercapnic ventilatory response (HCVR) was calculated as the change in ventilation per increase of five mmHg PetCO_2_ (Δ $\overset{\cdot }{\text{V}}$i/Δ PetCO_2_). The hypercapnic HVR was determined in the final five minutes of the protocol, which examined the difference in $\overset{\cdot }{\text{V}}$i from the isocapnic, normoxic stimulus to a combined hypoxic and hypercapnic stimulus. In all cases, the average $\overset{\cdot }{\text{V}}$i was obtained during a minimum 30-second period of the final minute of the normoxic and hypoxic conditions. $\overset{\cdot }{\text{V}}$i was standardized to barometric pressure at sea level, body temperature, and water vapor pressure (BTPS). For calculations of PetCO_2_, values of expired CO_2_ were measured and corrected with the corresponding barometric pressure to express them as partial pressure of CO_2_ in mmHg.

Heart rate (HR) was recorded from the pulse oximetry device throughout the entire protocol to measure HR responses to hypoxia, hypercapnia, and the combined hypoxia-hypercapnia stimuli. The average HR was obtained from the same minimum 30-s period as the average $\overset{\cdot }{\text{V}}$i.

#### Blood pressure measurements

Blood pressure measurements were taken either manually with a manual monitor or by an electric cuff monitor on the participant’s right arm while they were in a seated, relaxed position. Three separate measurements were taken during each session and up to three sessions of blood pressure measurements were taken per day at high altitude. Only one session of blood pressure measurements was collected at sea level.

#### Heart rate variability measurements

Estimation of sympathetic nervous system activity was approached through HR variability analysis of R-R peaks during a 10-minute period of electrocardiogram (lead I) obtained during resting conditions. Recording and analyses were performed at sea level and at high altitude using LabChart (Pro version 8.1.25, ADInstruments, Dunedin, New Zealand) using the default parameters indicated for humans, which includes the standard deviation of average R-R values (300s) and frequency bands of very low frequency (VLF: 0–0.04 Hz), low frequency (LF: 0.04–0.15), and high frequency (HF: 0.15–0.45). From the time-domain analysis, the standard deviation of R-R intervals (SDRR) and the root-mean-square value of successive differences between normal heart beats (RMSDD) was obtained. For frequency domain analysis, we measured power of LF and HF in normal units (nu) and the calculated LF/HF ratio.

#### Fasting glucose measurements

Fasting glucose measurements were collected by Metene TD-4116 Blood Glucose Monitor at sea level in the morning before departure to Barcroft Station and every morning thereafter at Barcroft Station. Participants were fasting for all measurements.

### Quantification and statistical analysis

We hypothesized O_2_ saturation would be greater in participants who received the probiotic compared to those who received the placebo. Based on a preliminary, unpublished study that compared SpO_2_ in two participants who received the probiotic versus two participants who received a placebo in an air cabin pressurized to the equivalent of approximately 8,000 feet during commercial flight, a sample size of 17 observations were required for an effect size of 0.8% SpO_2_ and 39 observations were required for an effect size of 0.5% SpO_2_. Both of these calculations were conducted for a Type I error rate of 5% and power of 80% in XLSTAT version 19.2.2 using the non-central Student distribution with non-centrality parameter. The effects of treatment group (probiotic or placebo) and timepoint on daytime SpO_2_ and mean nocturnal SpO_2_ were determined by two-way analysis of variance (ANOVA) with a Bonferroni *post hoc* test. Mixed-effects models to predict daytime SpO_2_ were completed utilizing the package lme4^96^ (Version 1.1.35.1) in RStudio (Version 2023.6.0.421). To account for the contribution of ventilation to SpO_2_, we interchangeably considered HVR on the second day at high altitude, HVR on the third day at high altitude, HVR at sea level, and minute ventilation at room air at high altitude in various models and selected the model with the lowest restricted maximum likelihood (REML) and lowest Akaike information criterion (AIC). We considered the interaction term “Treatment Group ∗ HVR on the second day at high altitude” within the model but dropped the term after it was shown to be an insignificant predictor of SpO_2_. Model assumptions were checked with package DHARMa (R package version 0.4.6). The ultimate mixed-effects model predicting daytime SpO_2_ included Treatment Group and HVR on the second day at high altitude as covariates with age, sex, and BMI as fixed effects, and participant and timepoint as random effects. The effects of treatment group and timepoint on secondary measurements were determined by two-way ANOVA and with a Bonferroni *post hoc* when appropriate.

### Additional resources

The study was approved by the UC San Diego Institutional Review Board (Protocol #807971) and registered on clinicaltrials.gov (Protocol ID NCT06552806).
