# Supplementary material for: Improved oxygen saturation and acclimatization with bacteriotherapy at high altitude
Source: iScience. 2025 Feb 17;28(4):112053. doi: 10.1016/j.isci.2025.112053 (PMC12022639; doi:10.1016/j.isci.2025.112053)
Supplement: Document S1. Figures S1, S2 and Table S1 [file mmc1.pdf]

iScience, Volume *28*

Supplemental information

Improved oxygen saturation and acclimatization with bacteriotherapy at high altitude

James J. Yu, Esteban A. Moya, Hunter Cheng, Kiana Kaya, Tim Ochoa, Santiago Fassardi, Eli Gruenberg, Alex Spenceley, Pamela DeYoung, Elizabeth V. Young, Laura A. Barnes, Alina Lugo, Ana Sanchez-Azofra, Jeremy E. Orr, Erica C. Heinrich, Atul Malhotra, and Tatum S. Simonson

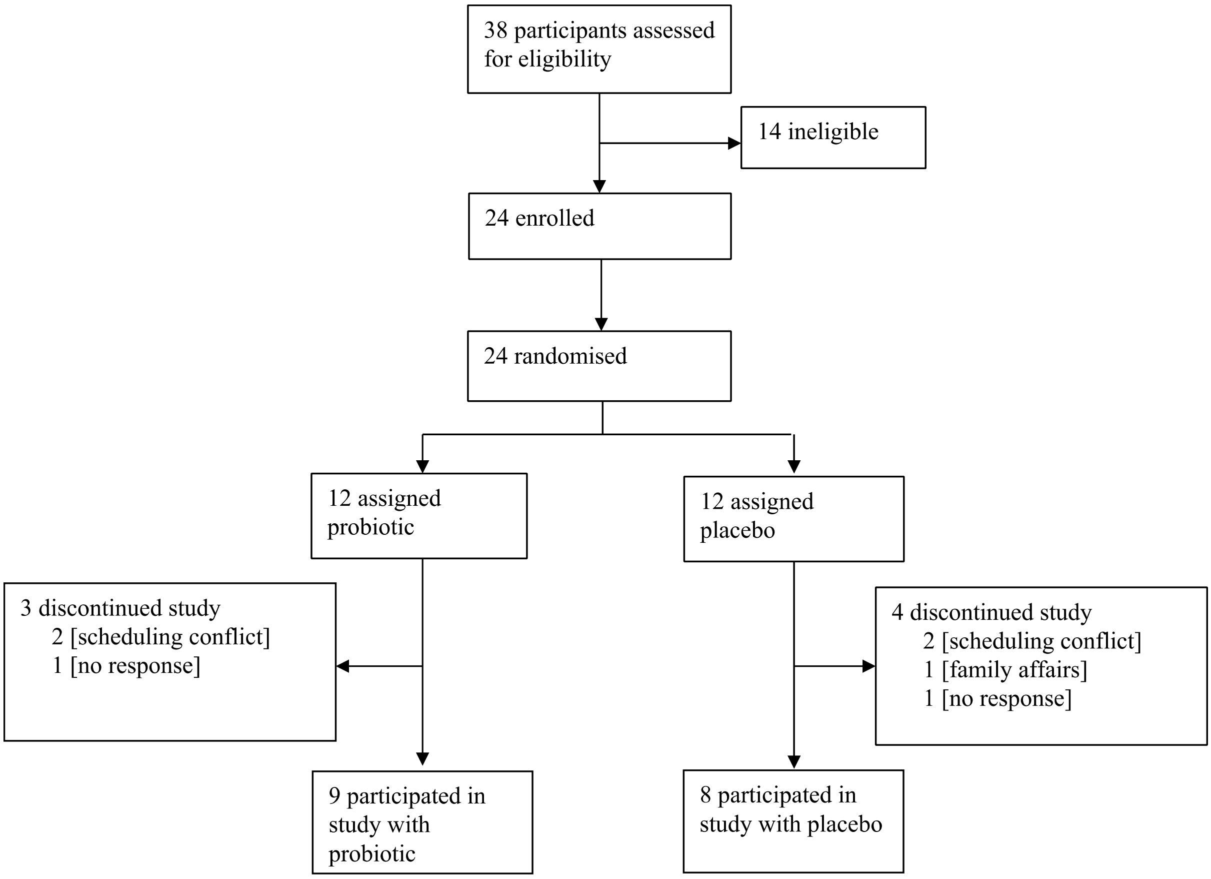

Supplementary Figure 1. Trial profile overview. 24 participants were enrolled in the study from 38 individuals that were assessed for eligibility. These 24 participants were randomly split into the probiotic group and the placebo group. 17 of these participants completed the study.

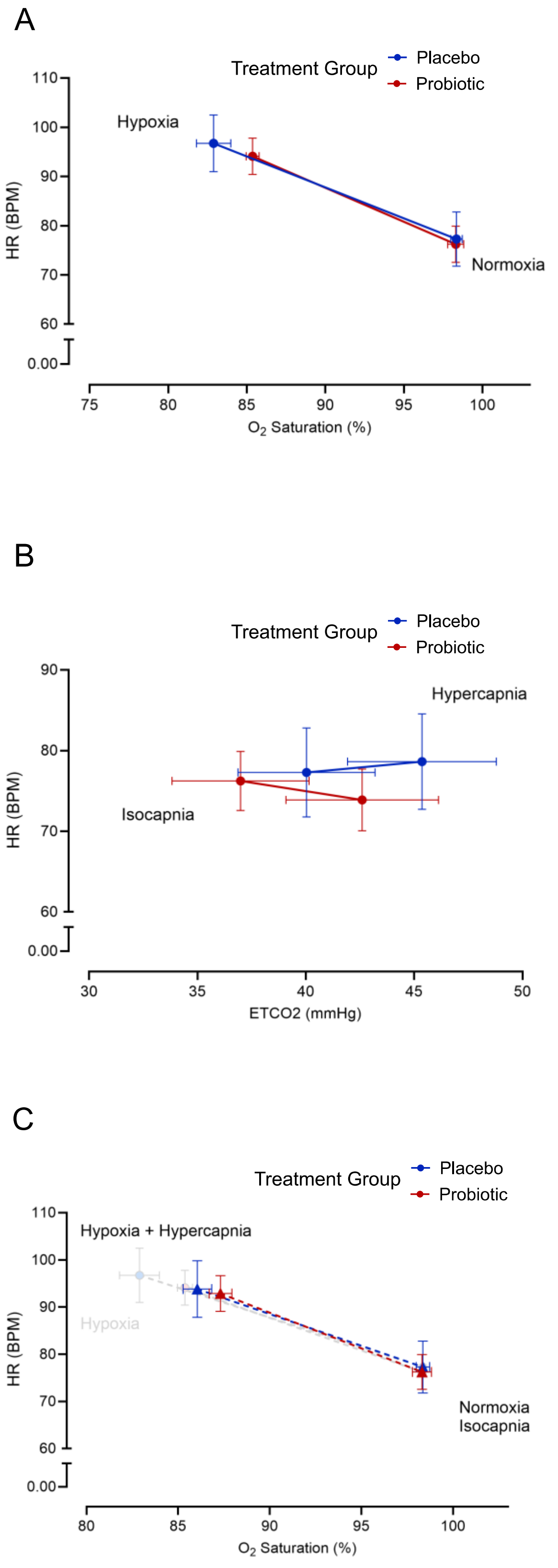

Supplementary Figure 2. Heart rate responses did not differ between treatment groups during ventilatory measurements in various hypoxic and hypercapnic stages. Heart rate responses did not differ during (A) hypoxia, (B) hypercapnia, and (C) combined hypercapnia-hypoxia between the treatment groups (two-way ANOVA) . All error bars represent SE.

Supplementary Table 1. Statistical summary of mixed effects model predicting daytime SpO_2 at high altitude.

effect	group	term	estimate	std.error	statistic
fixed		(Intercept)	81.94435406	3.87285291	21.1586538
fixed		Treatment_Probiotic	2.51026390	1.08636442	2.3107015
fixed		Ventilation_HVR	4.88064473	2.00383076	2.4356572
fixed		Age	-0.08142088	0.04632795	-1.7574893
fixed		Sex_M	1.14314393	1.40353518	0.8144747
fixed		BMI	0.15270351	0.12315996	1.2398795
ran_pars	ID	sd__(Intercept)	1.64274963		
ran_pars	Timepoint	sd__(Intercept)	0.67805624		
ran_pars	Residual	sd__Observation	2.57816016		

HVR, hypoxic ventilatory response; M, male; BMI, body mass index; SpO_2, oxygen saturation measured by pulse oximetry.
